# American Medical Association

**Published:** 1875-04

**Authors:** 


### Editorial.

American Medical Association.—The twenty-sixth annual session of this body will be held in the city of Louisville, Kentucky, on Tuesday, May 4th. The place of meeting is within easy reach by rail of the medical men of the South generally, and we trust there will be a large representation of the Southern profession. If the "representative men" who domicil in the cities of the East see fit to continue to give this national body the cold shoulder, let the physicians of the South at least join with the West in giving it an earnest and hearty support.

